# Dielectric Properties of Materials Used for Microwave-Based NO_x_ Gas Dosimeters

**DOI:** 10.3390/s24092951

**Published:** 2024-05-06

**Authors:** Stefanie Walter, Johanna Baumgärtner, Gunter Hagen, Daniela Schönauer-Kamin, Jaroslaw Kita, Ralf Moos

**Affiliations:** Department of Functional Materials, University of Bayreuth, 95447 Bayreuth, Germany

**Keywords:** dosimeter, radio frequency (RF), LTCC, NO_x_, gas sensor, Microwave Cavity Perturbation, dielectric properties

## Abstract

Nitrogen oxides (NO_x_), primarily generated from combustion processes, pose significant health and environmental risks. To improve the coordination of measures against excessive NO_x_ emissions, it is necessary to effectively monitor ambient NO_x_ concentrations, which requires the development of precise and cost-efficient detection methods. This study focuses on developing a microwave- or radio frequency (RF)-based gas dosimeter for NO_x_ detection and addresses the optimization of the dosimeter design by examining the dielectric properties of LTCC-based (Low-Temperature Co-fired Ceramics) sensor substrates and barium-based NO_x_ storage materials. The measurements taken utilizing the Microwave Cavity Perturbation (MCP) method revealed that these materials exhibit more pronounced changes in dielectric losses when storing NO_x_ at elevated temperatures. Consequently, operating such a dosimeter at high temperatures (above 300 °C) is recommended to maximize the sensor signal. To evaluate their high-temperature applicability, LTCC substrates were analyzed by measuring their dielectric losses at temperatures up to 600 °C. In terms of NO_x_ storage materials, coating barium on high-surface-area alumina resolved issues related to limited NO_x_ adsorption in pure barium carbonate powders. Additionally, the adsorption of both NO and NO_2_ was enabled by the application of a platinum catalyst. The change in dielectric losses, which provides the main signal for an RF-based gas dosimeter, only depends on the stored amount of NO_x_ and not on the specific type of nitrogen oxide. Although the change in dielectric losses increases with the temperature, the maximum storage capacity of the material decreases significantly. In addition, at temperatures above 350 °C, NO_x_ is mostly weakly bound, so it will desorb in the absence of NO_x_. Therefore, in the future development of a reliable RF-based NO_x_ dosimeter, the trade-off between the sensor signal strength and adsorption behavior must be addressed.

## 1. Introduction

Nitrogen monoxide (NO) and nitrogen dioxide (NO_2_) are byproducts from the combustion of fossil fuels or renewable resources. These nitrogen oxides (NO_x_) have been identified as a direct threat to human health and the environment. NO_x_ emissions consist primarily of NO, which is subsequently oxidized in the atmosphere to form the more harmful NO_2_. To protect human health, a 1-h limit value for a NO_2_ concentration of 200 μg/m^3^ (or about 105 ppb) was introduced in the European Union in 2008 (Directive 2008/50/EC) [1]. This threshold may not be exceeded more than 18 times in a given calendar year. Additionally, an annual limit value of 40 μg/m^3^ (about 21 ppb) has been set, with 30 μg/m^3^ being considered critical for vegetation. In Germany, approximately 500 automated measuring stations [2], such as chemiluminescence detectors (CLDs) [3], monitor NO_2_ concentrations to ensure air quality. However, conventional gas analysis systems are both bulky and expensive. Furthermore, conducting an accurate assessment of compliance with limit values over long periods of time, particularly at low concentrations, is challenging due to zero-point drifts, non-linearity, and signal noise [4,5,6]. Conversely, gas dosimeters that rely on the diffusion-limited enrichment of a sensitive material are well suited for determining the average value of exposure over a given period [7]. Developing more cost-efficient and accurate gas analysis devices would greatly contribute to monitoring the impact of nitrogen oxide on our ecosystem.

For this purpose, a dosimeter capable of measuring average NO_x_ concentrations over long time periods is being developed. Previous dosimeters for such applications were often based on measuring the resistivity of a sensitive material, such as potassium or manganese [7]. However, the choice of possible materials is limited to those with high electrical conductivity. For instance, the use of barium carbonate (BaCO_3_) in resistive NO_x_ dosimetry is hindered by its thermally activated electrical conductivity that is six orders of magnitude lower compared to that of potassium carbonate (K_2_CO_3_), which, in turn, has to be stabilized due to its hygroscopic sensitivity on electrical properties [8,9]. An emerging alternative is the use of microwaves, i.e., radio frequency (RF) electromagnetic waves, to detect a change in the dielectric properties of a gas-sensitive material. As studies on barium-based catalysts have shown, RF sensors can be used to deduce the amount of stored NO_x_ in NO_x_ storage catalysts [10]. However, such a system must be miniaturized for application in indoor air monitoring. Therefore, many planar microwave gas sensors have been investigated in recent years [11,12,13,14,15,16,17,18,19,20,21,22,23]. Studies have analyzed how a sensitive material affects the propagation of electromagnetic waves around transmission lines, such as microstrips or striplines. To detect this effect, the material is often applied in the area of a resonance structure, which allows the sensor to be evaluated in terms of the resonant frequency or quality factor.

Our study aims to optimize the design of such an RF-based dosimeter by analyzing the dielectric properties of different sensor substrates that are suitable for high-temperature applications as well as the properties of barium-based NO_x_ storage materials during the storage of nitrogen oxides, building on the initial analyses presented in [24]. This research is crucial for the further development of efficient and reliable NO_x_ monitoring systems, which are essential for environmental protection and public health.

## 2. The Working Principle of the RF-Based NO_x_ Gas Dosimeter

To gain a deeper understanding of the parameters to be considered in the development of a planar RF dosimeter, this section explains the fundamental operating principle of such a sensor.

Unlike conventional gas sensors, gas dosimeters do not measure the analyte concentration, but rather the amount of analyte that has accumulated since the start of the measurement cycle. Since even the smallest analyte concentrations contribute to the sensor signal change, gas dosimeters are particularly suitable for the long-term detection of highly diluted analytes, e.g., for the monitoring of air quality limits, especially since immission limits are also given as doses [7]. The measured dose *D* depends on the time-varying gas concentration *c*(*t*) according to Equation (1).

(1) $$D=\int_{0}^{t_{end}}c(t)dt$$

**Figure 1:**
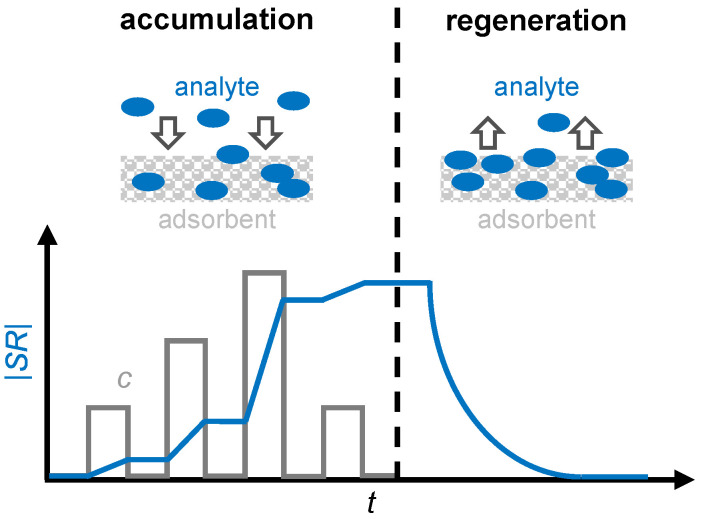
The operating principle of a gas dosimeter with a sensitive layer as an adsorbent: the scheme and sensor response |*SR*| (blue) in correlation with the analyte concentration *c* (grey) for the accumulation and regeneration period. Adapted from [7].

In addition, by differentiating the dosimeter signal, it is also possible to draw conclusions about the gas concentration [25]. Figure 1 illustrates the operating principle of a gas dosimeter using a sensitive layer. Suitable sensitive materials are adsorbers that incorporate analyte molecules and change at least one physical property that can be measured as a sensor signal, such as electrical resistance or permittivity, as the accumulation progresses. However, since the amount of adsorbed gas in a sensitive material is limited, a regeneration cycle must be conducted periodically to desorb the gas to be detected [26]. This desorption can be accomplished thermally, for example, by heating the sensor and the sensitive material to a temperature at which the analyte gas cannot be permanently stored. Zeolites, for example, exhibit this type of behavior when storing ammonia [27], as well as barium-based materials in automotive catalysts during NO_x_ storage [28].

To dosimetrically measure NO_x_ using an RF sensor, the dielectric properties of the storage material have to change depending on the amount of stored NO_x_. In practice, the dielectric properties of a material can only be measured by placing it on a planar transmission line, while the use of a resonance method allows for higher accuracy and sensitivity. From the characteristic parameters of the excited resonance mode (resonant frequency and/or quality factor), the dielectric properties of the sensitive material and, thus, the dosimeter signal, can be derived.

A simple form of such a planar resonance structure can be a ring coupled through an air gap to a straight excitation line, which is then connected to a network analyzer. This can then generate standing electromagnetic waves on the ring structure at certain frequencies, which depend on the characteristic length $L_{\mathrm{ch}}$ of the ring, which is approximately its mean circumference and the effective permittivity $\varepsilon_{\mathrm{eff}}$ of the surrounding material, as described in Equation (2) [29]. Besides the fundamental resonance mode, higher ones can also be excited at multiple *n* of its frequency.

(2) $$f_{\mathrm{res}}=n\cdot \frac{c}{L_{\mathrm{ch}}\sqrt{\varepsilon_{\mathrm{eff}}}}$$

**Figure 2:**
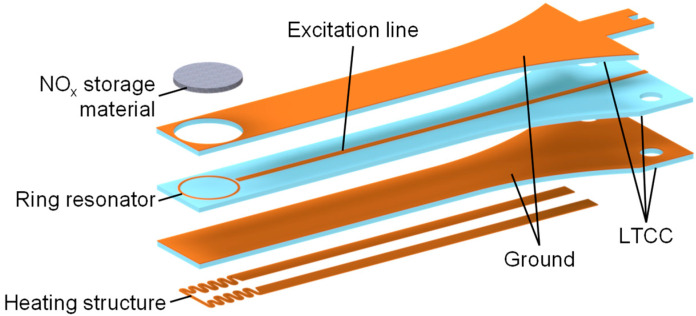
A schematic setup of the planar RF-based NO_x_ dosimeter with a substrate length of 48.0 mm, a thickness of 1.5 mm, and a width of 6.3 mm at the front (the width increases to 14.0 mm at the mounting location). The heating structure is not the topic of this paper and will be presented in detail in future publications. Adapted from [24].

The materials characterized in this paper will be used for upcoming measurements in a multilayer sensor setup, similar to that shown in Figure 2, using a simple ring resonance structure. To achieve a high sensor signal, a thickness of several hundred micrometers for the sensitive layer is advisable. The reason for this is the dependence of the resonant frequency on the effective complex permittivity (Equation (3)), which, in turn, is determined by the dielectric properties of the materials surrounding the conductor and the electric field there [30].

(3) $${\varepsilon =\varepsilon }^{\prime }-j\varepsilon^{\prime \prime }$$

For the realization of such a structure, it is possible to obtain different configurations of the transmission line. The calculations of the line losses shown in this paper are based on the stripline configuration, which is defined by a conductor track shielded on both sides by a ground plane. An alternative is to use a coated microstrip configuration with no ground at the top of the sensor [31]. In the case of the stripline, the effective permittivity used to design the transmission line is equal to the substrate permittivity, allowing for a simple calculation of the optimal line width. In the previous microstrip design, determining the geometric properties of the conductor required the consideration of additional effects such as dispersion [12].

Nevertheless, for both transmission lines, their width cannot be chosen arbitrarily, since it determines the characteristic impedance in conjunction with the dielectric properties of the substrate and the sensor material [32]. A change in the width can thereby influence the coupling of the resonance structure and, thus, the quality of the resonance [33].

In addition, the substrate must be suitable with losses that are low enough to allow for the excitation of an evaluable resonance signal. For this reason, the dielectric properties of the material used as the sensor substrate must be known for the dimensioning of the dosimeter sensor. In addition, a heating structure will be integrated to adjust the temperature of the storage material. This will allow for regeneration through the thermal desorption of the adsorbed gas and can enhance the adsorption characteristics of the storage material, which are often temperature-dependent. Its adsorption and desorption behaviors are also investigated in this study to precisely design a heater for the optimal temperature range at a later stage.

## 3. Development of Sensor Materials

### 3.1. Sensor Substrate

The change in the sensor design compared to [34] lies in a multilayer substrate structure. Due to the easier processability, LTCC (Low-Temperature Co-fired Ceramic) is used as the sensor substrate instead of alumina.

In addition to DuPont GreenTape 951 [35], which is frequently used in sensor development, low-loss DuPont GreenTape 9K7 [36], which was specially developed for high-frequency applications, is also examined in this work regarding its suitability for the dosimeter to be developed. To ensure the sufficient stability of the sensor, it will consist of a total of four layers, each with a thickness of 254 μm.

**Figure 3:**
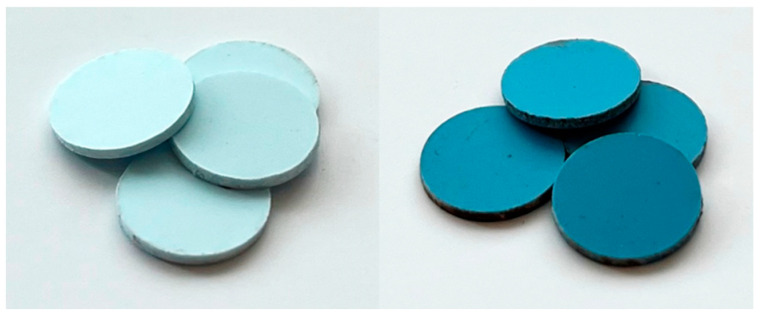
LTCC materials DuPont 9K7 (**left**) and 951 (**right**). Manufactured platelets that were used to measure dielectric properties and shrinkage behavior.

Therefore, in order to determine the shrinkage characteristics for the oven to be used for sensor production, platelets were fabricated that, like the finished sensor, consist of four layers and have a diameter of 10 mm in the unfired state. They are shown in Figure 3. These samples were also used to determine the dielectric properties of the LTCC in Section 4.1, whereby 20 stacked platelets were measured together to increase the accuracy of the used measurement system, as described in Section 3.3. The dimensions of the platelets were measured after the firing process on a total of 30 platelets. This resulted in the shrinkage values shown in Table 1, which are within the shrinkage tolerance of the data sheet values.

### 3.2. NO_x_ Storage Materials

For an RF dosimeter to detect the NO_x_ loading of its storage material, it must change its permittivity or its dielectric losses. This change occurs in barium-based materials due to the conversion of carbonate to nitrate during NO_x_ storage. These materials are frequently used in automotive storage catalysts [37,38,39,40], where previous measurements with an RF-based system have already shown that NO_x_ storage can be easily detected [10], which is why they shall be used to develop the sensor materials.

Nitrogen oxides are stored in barium-based materials by adsorbing NO_2_ in an oxidizing environment by releasing CO_2_ (Equation (4)) [41,42].

(4) $${\mathrm{BaCO}}_{3}+2\;{\mathrm{NO}}_{2}+\text{½}\;O_{2}⟷\;\mathrm{Ba}{\left({\mathrm{NO}}_{3}\right)}_{2}+{\mathrm{CO}}_{2}$$

However, incorporation and, therefore, the possibility to detect the current pollutant dose is not limited to NO_2_. It should also be possible to detect NO. Therefore, it is necessary to oxidize it to NO_2_, which can be achieved at catalytically active platinum sites on the surface of the storage material (Equation (5)) [40,42,43,44]. For a sufficiently fast oxidation of NO, however, a sensor temperature of at least 200 °C is required [45].

(5) $$2\;\mathrm{NO}+{\;O}_{2}\to \;2{\;\mathrm{NO}}_{2}$$

The formed nitrides decompose at higher temperatures, allowing the sensor material to be regenerated [7,46]. A decrease in the storage capacity is typically observed above 300 °C [47]. According to Equation (4), CO_2_ must be present to restore the carbonate state of the storage material when releasing nitrogen oxides. Nevertheless, even in CO_2_-free atmospheres, nitrogen oxides will still be released. However, the storage material will not be present as a carbonate but as hydroxide (e.g., Ba(OH)_2_) or oxide (e.g., BaO) [46,48].

**Figure 4:**
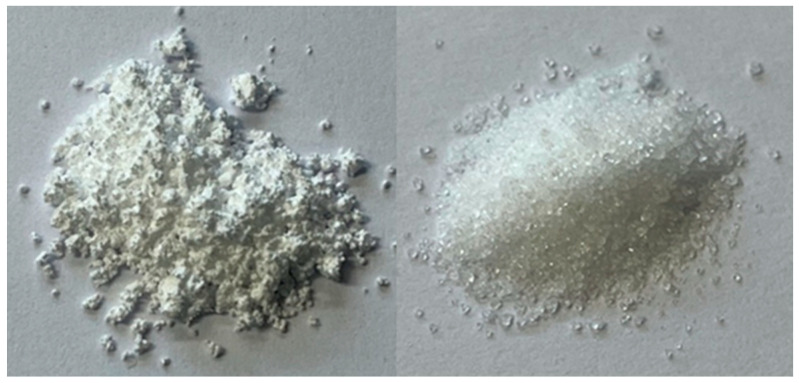
Photograph of analyzed barium carbonate (**left**) and barium nitrate (**right**).

Since our goal is to develop an air quality sensor, the dielectric properties of the sensor material in the “unloaded” and “loaded” states must be known in order to estimate the potential sensor signal. For this purpose, the material properties of pure barium carbonate (BaCO_3_) and barium nitrate (Ba(NO_3_)_2_) (both supplied by Merck and with a purity of >99%, shown in Figure 4) were first determined in this paper.

For the preparation of a NO_x_ dosimeter material allowing for high storage rates as well as a high absolute storage quantity, a high surface area lanthanum-stabilized γ-alumina (Sasol Puralox SCFa-140 L3) was chosen, onto which a barium coating was applied. This was carried out by infiltrating the carrier substrate with barium acetate Ba(CH_3_COO)_2_ (also supplied by Merck and with a purity of >99%) dissolved in water. After drying the powder at 110 °C, it was calcined at 550 °C for four hours in air, forming a barium carbonate due to the CO_2_ in the atmosphere [46]. In this study, powders with three different loadings were prepared, corresponding to barium carbonate weight percentages of 5.6 wt.%, 11.2 wt.%, and 16.9 wt.%, respectively.

These powders were analyzed regarding their Brunauer–Emmett–Teller (BET) surface area (Table 2). Pure BaCO_3_ has a notably lower surface area compared to all barium-coated alumina samples by a factor of more than 25. Compared to the uncoated alumina, however, the surface area decreased with an increasing barium carbonate content. Nevertheless, the sample with the highest barium carbonate coating of 16.9 wt.% was selected for the investigations regarding the dielectric behavior. Since the available BET surface area was only slightly reduced compared to the other barium carbonate loadings (−27% compared to the 5.6 wt.% and −10% compared to the 11.2 wt.% sample), the higher absolute loading may have led to a higher storage capability, which could have resulted in a more pronounced change in the dielectric properties. However, for the dosimeter application, it should be noted that a faster NO_x_ storage due to a larger surface area being available for adsorption could have increased the sensitivity to low NO_x_ concentrations, and therefore, a different BaCO_3_ loading may be preferable for further sensor development.

To ensure that the sensor material was not only sensitive to NO_2_ but also to NO, the oxidation reactions needed to be catalytically enhanced. For this purpose, platinum was deposited on the dosimeter material. This was realized by adding tetraamine platinum(II) chloride dissolved in water (supplied by Merck; the pure platinum content is approx. 55 wt.% according to the manufacturer) to the powder in an amount of 1 wt.% relative to the mass of the barium-coated powder. After renewed drying, this was reduced to elemental platinum by a two-hour annealing process at 500 °C under forming gas (5% H_2_ in N_2_). Consequently, the elemental platinum loading was approx. 0.6 wt.%. The weight percentage of barium carbonate remained almost unchanged due to the small amount of platinum.

### 3.3. The Determination of Dielectric Properties Using the MCP Method

To achieve an optimized signal sensitivity, it was necessary to design the geometry of the resonance structure based on the dielectric properties of the LTCC substrate and the barium-based NO_x_ storage material. These properties can be determined by the Microwave Cavity Perturbation (MCP) method using a setup described earlier by Dietrich et al. [49], as explained in detail by Steiner et al. when measuring porous powder samples [50]. In the MCP method, a sample is placed within a cylindrical cavity that allows for the excitation of electromagnetic resonances. Due to the dielectric properties of the sample, a shift in the resonant frequency Δf and in the inverse quality factor $\Delta \left(1/Q\right)$ occurs. These shifts can then be utilized, according to Equations (6) and (7), to derive the permittivity $\varepsilon^{\prime }$ and the dielectric losses $\varepsilon^{\prime \prime }$, which are composed of polarization and conductivity losses, of the material sample.

(6) $$\Delta f\sim \left(\varepsilon^{\prime }-1\right)$$

(7) $$\Delta \left(\frac{1}{Q}\right)\sim \varepsilon^{\prime \prime }$$

These correlations are only valid if the electromagnetic field within the resonator is minimally disturbed by the introduction of the sample. For samples with large volumes or high dielectric losses, this condition is not met. Nonetheless, it is possible to account for these perturbations by implementing corrections. The correction in [50], which addresses depolarization behavior and field distribution, allowed, for example, a precise determination of the effective properties of ceria–zirconia powders [51]. In cases where the samples are porous, the properties of the bulk material can be determined by considering the influence of air on the effective material properties using mixing rules [52,53].

For the determination of the electric properties of the LTCC samples, the application of mixing rules was not necessary since the samples were dense. However, all examined NO_x_ storage materials were in powder form with an air content of around 90 vol.%. This was determined by comparing the bulk volume inside the MCP setup with the particle volume measured with a gas pycnometer (Micromeritics AccuPyc 1330). The amount of powder introduced into the MCP setup was 290 mg for the coated barium samples, and a larger sample quantity of 665 mg was chosen for the pure barium carbonate samples in order to increase the accuracy of the measurement procedure, despite resulting in longer adsorption times, since only a small change in the dielectric properties was expected due to the expected limited NO_x_ storage behavior. To derive the bulk properties of the samples, we employed the Wiener mixing rule, which assumes a linear relationship between the air fraction and the dielectric properties [53,54]. Although it is unknown whether this mixing rule actually applies to the measured materials, it still allows for a comparison of their storage and release behaviors as all samples had almost the same air content.

The used measurement setup, as described in detail in [49], allows for the dielectric properties of samples to be determined at temperatures up to 600 °C. The sample temperature *T* is determined by calculating the average value obtained from two type K thermocouples. These thermocouples are placed inside the sample tube but outside the resonator cavity to avoid interference with the resonance. Additionally, the NO_x_ storage materials can be exposed to different gas atmospheres over the entire temperature range, allowing for measurements to be taken during both NO_x_ storage and regeneration. The barium acetate-based materials were flushed with a constant total flow rate of 500 mL/min. For the pure barium carbonate, a slightly lower flow rate of 400 mL/min was set to prevent the fluidization of the powder sample. Mass flow controllers (MFCs) were used to create an atmosphere of 20 vol.% oxygen in nitrogen. In addition, adjustable concentrations of NO and NO_2_ can be added to investigate the storage capacity of the material. To then enable the desorption of the previously stored NO_x_, the powder was heated to over 550 °C, which will also be achieved in the sensor setup being developed.

To determine the dielectric properties, the parameters of the resonance mode TM_010_ (located at a frequency of approximately 1.18 GHz) were analyzed. Therefore, the S-parameter *S*_21_ was measured using a vector network analyzer (VNA, MS46322B, Anritsu, Atsugi, Kanagawa Prefecture, Japan) and evaluated in terms of the resonant frequency and quality factor using the methods described in [55,56,57]. A calibration measurement with an empty sample tube was performed prior to each material sample. This step is necessary because the MCP method uses the resonant parameter shift caused by the insertion of the materials to determine their dielectric properties.

## 4. Dielectric Properties of Sensor Materials

### 4.1. LTCC Substrate

To verify the suitability of the substrate materials for their application in a high temperature sensor, the two different types of LTCC (DuPont 951 and DuPont 9K7) were analyzed regarding the impacts of their dielectric losses on the transmission behavior of the striplines used for the RF dosimeter. To minimize the attenuation of the measured resonance mode within the temperature range preferred for NO_x_ storage, it is recommended to use a substrate with lower losses. The loss factor tan⁡δ describes the ratio of $\varepsilon^{\prime \prime }$ to $\varepsilon^{\prime }$. According to the manufacturer’s data sheet [36], the 9K7 is designed for high-frequency applications and has a low loss factor of only 0.001 at 10 GHz compared to 0.014 for the DuPont 951. It is important to note that these values are specific to room temperature. Knowing the temperature-dependent behaviors of the material properties is crucial for operating the sensor over a wide temperature range.

**Figure 5:**
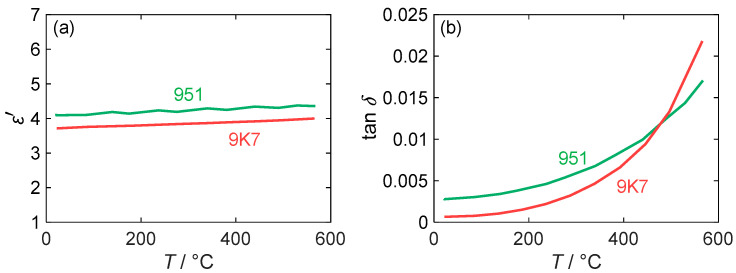
(**a**) Permittivity $\varepsilon^{\prime }$ and (**b**) loss factor tan⁡δ of different LTCC materials measured at 1.18 GHz depending on substrate temperature (red: DuPont 9K7; green: DuPont 951). Adapted from [24].

The values for the real part of the permittivity $\varepsilon^{\prime }$ and the loss factor, tan⁡δ, were previously measured by the MCP method in [24] and are shown in Figure 5 in a temperature range from 20 to 600 °C. Regarding the permittivity, the measured values for both LTCC materials are almost independent of its temperature and vary between 3.6 and 4.0 for 9K7 and between 4.1 and 4.4 for 951. The DuPont 951 therefore has a higher dielectric constant than the 9K7, as indicated in the data sheet. However, the measured absolute values are significantly lower than the data sheet values. A possible reason for this discrepancy could be dispersion effects, as the data sheet values are given for a higher frequency of 10 GHz. While dispersion effects could also affect dielectric losses, the measured data reveal a strong dependence on temperature, as is typical for ceramic materials. The losses of both LTCC materials increase non-linearly with the temperature, but at different rates. Contrary to its intended use, DuPont 9K7 exhibits higher dielectric losses than DuPont 951 at temperatures exceeding 500 °C. At room temperature, the data sheet value for DuPont 951 (at a frequency of 10 GHz) is 0.014, which is substantially above the measured value of 0.0028. For 9K7, the data sheet value of 0.001 is close to the experimental value of 0.0007. Considering the scope of the RF dosimeter, which is primarily intended to operate within a temperature range of 200 to 400 °C (cf. Section 4.2), DuPont 9K7 is still the preferred substrate for this application due to its lower losses in the temperature range.

**Figure 6:**
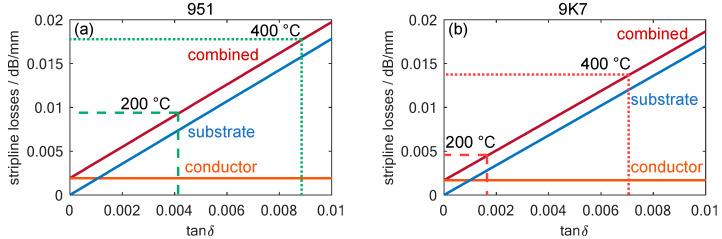
The calculated transmission losses for a stripline with a four-layer LTCC substrate in the fired state as a function of the loss factor, tan⁡δ, of a substrate with (**a**) $\varepsilon^{\prime }=7.8$, which is the data sheet value for the 951 LTCC at 10 GHz and room temperature and (**b**) $\varepsilon^{\prime }=7.1$, which is the data sheet value for the 9K7 LTCC at 10 GHz and room temperature. The measured loss factors for DuPont 951 and 9K7 at temperatures of 200 and 400 °C are also marked.

To further evaluate the impact of substrate losses on stripline transmission characteristics, their total losses were theoretically calculated using the equations given in [58]. The total losses also depend, to a small extent, on the permittivity of the substrate. As the sensor to be developed is intended to operate at frequencies close to the 10 GHz value of the data sheet permittivity, the data sheet values were used for the theoretical calculations instead of the measured values. Figure 6 illustrates these losses calculated for both examined substrates, based on their loss factors, and highlights the previously measured loss values for the substrates at 200 and 400 °C. However, transmission line losses originate not only from dielectric losses within the substrate, but also from the limited conductivity of the stripline conductor itself. These losses can also be calculated theoretically and are shown in Figure 6.

In the NO_x_ dosimeter to be developed, the losses were calculated for the case of four LTCC layers surrounding the stripline, which was dimensioned for a wave impedance of 50 Ω. The transmission line was assumed to be screen-printed using the recommended gold-based conductor, DuPont LL505. The losses caused by this effect can be calculated based on the data sheet value for its minimum conductivity of 5 Ω/□.

The resulting losses not only depend on the loss factor, tan⁡δ, of the substrates, but also on permittivity and geometry, which are different for both substrates due to their shrinkage behavior and their compositions. Therefore, the calculated values for DuPont 951 and 9K7 are slightly different. However, the influence of the substrate loss factor remains the primary influence. Moreover, the calculations reveal that conductor losses only slightly contribute to the total losses of the transmission line in the temperature ranges in which the sensor operates, even with the lower loss of 9K7. A comparison of the two LTCC films shows that at a temperature of 200 °C, the 951 has twice the transmission losses, and at 400 °C, there are still losses higher than 40%. Thus, this theoretical analysis validates the choice of 9K7 LTCC for the desired NO_x_ gas dosimeter application.

### 4.2. Barium-Based Gas Dosimeter Material

#### 4.2.1. Pure Barium Carbonate

Following the examination of the LTCC-based sensor substrates, the dielectric behavior of the NO_x_-sensitive barium-based material is to be investigated. The first step is to analyze the complex permittivity range of the storage material with and without NO_x_ loading. For this purpose, the dielectric properties of barium carbonate (BaCO_3_) and barium nitrate (Ba(NO_3_)_2_) were measured from room temperature to 500 °C using the MCP method. In addition to the dielectric losses already shown in [24], the permittivity of both materials is now also given. Furthermore, although the loss values in [24] were obtained using a mixing rule with an exponential approach, the values are now calculated using the Wiener mixing rule, as described in Section 3.3.

**Figure 7:**
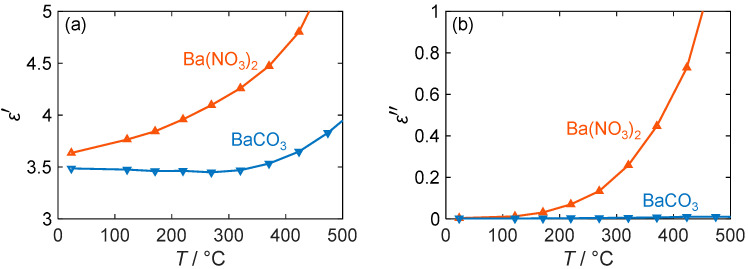
(**a**) Permittivity $\varepsilon^{\prime }$ and (**b**) dielectric losses $\varepsilon^{\prime \prime }$ of barium-based NO_x_ storage materials depending on their temperature *T* (blue: BaCO_3_; red: Ba(NO_3_)_2_).

As can be seen in Figure 7a, the permittivity of the two materials differs slightly. Nitrate generally exhibits higher polarization effects, although the difference is only marginal at room temperature. However, with an increasing temperature, the difference becomes more pronounced as the permittivity of nitrate rises from 3.6 to 4.6 until a temperature of 400 °C is reached, while the $\varepsilon^{\prime }$ value of carbonate remains almost unchanged at 3.5. Only at temperatures above 400 °C does the permittivity of barium carbonate also increase. Despite the small expected change due to the incorporation of NO_x_, such changes in permittivity can be easily detected with an RF-based gas dosimeter via the resonant frequency. For example, similar large permittivity changes occur in ammonia-adsorbing zeolites [59] and have been measured with the RF sensor used in [34]. However, this only applies if a large fraction of the barium carbonate is converted to nitrate when the storage material is exposed to NO_x_.

If this is not the case, the RF dosimeter can still evaluate the quality factor of its resonance. This resonance parameter mainly depends on the dielectric losses of the sensitive material and is therefore dependent on the present barium compound, especially at higher temperatures. Thus, Figure 7b shows that at room temperature, both nitrate and carbonate have almost equal losses of less than 0.01. However, the value of $\varepsilon^{\prime \prime }$ of BaCO_3_ increases only slightly with the temperature and remains below 0.05, while that of Ba(NO_3_)_2_ increases strongly (almost exponentially). Therefore, at 200 °C, the losses of barium nitrate are 8 times higher, and at 400 °C, they exceed those of barium carbonate by more than 20 times. Due to the significant difference between the two barium compounds, operating at high temperatures could result in a larger sensor response for the RF gas dosimeter. However, limitations such as the NO_x_ adsorption behavior must be taken into account, especially for dosimeter applications, as discussed in the following sections. In addition, the signal quality may decrease with an increasing temperature due to higher losses of the LTCC substrate, which will be the subject of further research.

As with permittivity, the changes in dielectric losses and the sensor signal strength depend on the storage capability of the material. However, for pure barium carbonate, the surface area is very small, as shown in Table 2. This poses a problem because nitrogen oxides are stored near the surface of the material [45,60]. Therefore, a larger surface area is advantageous for detecting the NO_x_ dose present with a sufficient change in complex permittivity.

Despite the small surface area of the barium carbonate, its storage capacity should be analyzed as a reference value for barium-coated materials. For this purpose, the powder was exposed to a concentration of 200 ppm NO_2_ at a constant sample temperature. No investigations were carried out on the storage behavior of NO, as the catalytic component necessary for oxidation to NO_2_ is not present in barium carbonate. Once the material was saturated, the NO_2_ dosing was first stopped to allow for the analysis of the desorption of weakly bound NO_x_, which was adsorbed due to the absence of NO_2_ partial pressure. Afterward, the remaining stored NO_x_ (strongly bound NO_x_) was removed by heating the sample to 550 °C.

**Figure 8:**
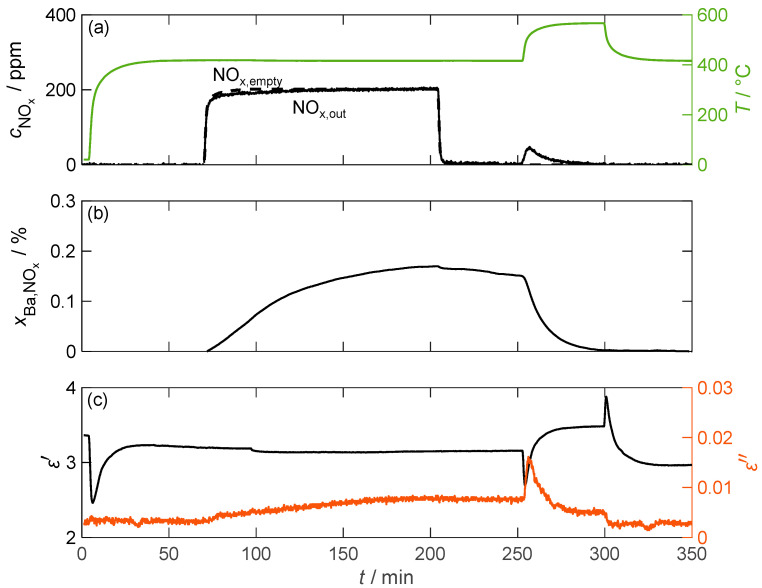
The storage and regeneration behavior of pure BaCO_3_ over time; (**a**) the NO_x_ concentration $c_{\mathrm{NOx}}$ without (dashed line) and with (solid line) the storage material measured by FTIR downstream of the MCP setup as well as the temperature *T* of the storage material (green line); (**b**) the storage utilization $x_{\mathrm{Ba},\mathrm{NOx}}$ calculated based on an integration of the measured nitrogen oxide concentration; (**c**) the permittivity $\varepsilon^{\prime }$ and dielectric losses $\varepsilon^{\prime \prime }$ of the storage material.

Figure 8 displays the temporal course of the experiment conducted at 417 °C. The NO_x_ concentration downstream of the barium carbonate was measured using FTIR ($c_{\mathrm{NOx},\mathrm{out}}$). In addition, the NO_x_ concentration was measured without a material sample to consider time delays in the concentration curve compared to the values dosed by the MFCs ($c_{\mathrm{NOx},\mathrm{empty}}$). The storage utilization $x_{\mathrm{Ba},\mathrm{NOx}}$ was calculated in an integrative manner from the resulting concentration difference related to the proportion of BaCO_3_ in the sample $n_{\mathrm{BaC}O_{3}}$ according to Equation (8). ${\dot{V}}_{\mathrm{Gas}}$ represents the total volume flow through the sample, and $V_{\mathrm{mol},\mathrm{Gas}}$ represents the molar volume of the gas.

(8) $$x_{\mathrm{Ba},\mathrm{NOx}}=\int_{0}^{t_{\mathrm{end}}}(c_{\mathrm{NOx},\mathrm{empty}}(t)-c_{\mathrm{NOx},\mathrm{out}}(t))\cdot \frac{{\dot{V}}_{\mathrm{Gas}}(t)}{V_{\mathrm{mol},\mathrm{Gas}}}\;dt\cdot \frac{1}{2\cdot n_{\mathrm{BaC}O_{3}}}$$

The above indicates which portion of the barium carbonate molecules was converted to nitrate due to NO_x_ incorporation. However, due to integration errors caused by deviations in the measured gas concentration of only a few ppm, the calculated storage utilization did not reach zero after the thermal desorption process. To correct this issue, a constant factor was applied to the measured NO_x,out_ concentration throughout the measurement sequence.

As expected, hardly any NO_x_ was stored due to the small surface area. A maximum of 0.17% of the barium carbonate was converted to nitrate. This low storage capacity is also reflected in the dielectric properties. No change in permittivity can be associated with NO_x_ storage. Larger changes in the measured $\varepsilon^{\prime }$ only occurred during temperature changes. However, this was most likely not due to a change in the material properties, but rather an incorrect determination of the resonant parameter shift as a result of a non-stationary temperature distribution within the MCP setup. The dielectric losses, on the other hand, increased by more than a factor of 2 from 0.0032 to 0.0079 between 70 min and 250 min. However, due to the small absolute losses, this change is only slightly pronounced compared to the signal noise. The thermal desorption of the stored NO_x_ can also be easily identified in the loss data. Due to the increased temperature, the dielectric losses initially increased by a factor of two. However, due to the desorption of NO_x_, the losses decreased rapidly and reached a stationary value after 30 min. When the sample temperature was lowered back to around 400 °C, the losses then returned to the pre-loading values.

**Figure 9:**
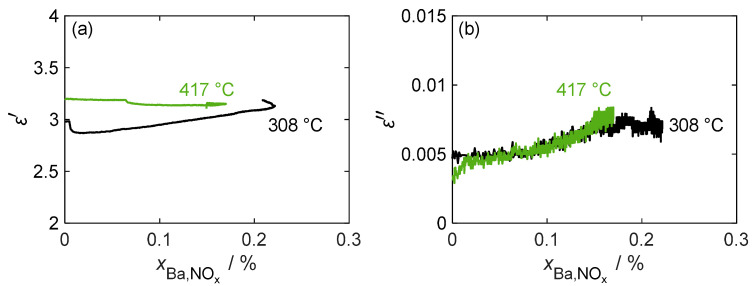
(**a**) The permittivity $\varepsilon^{\prime }$ and (**b**) dielectric losses $\varepsilon^{\prime \prime }$ of pure BaCO_3_ during the NO_x_ storage and the weakly bound desorption phases at different temperatures over the storage utilization period $x_{\mathrm{Ba},\mathrm{NOx}}$.

A more detailed analysis of the relationship between the complex permittivity and stored NO_x_ is provided in Figure 9. The figure includes an additional measurement at a lower temperature of 308 °C, which shows a slightly higher maximum storage capacity. However, the dielectric losses do not exceed those of the 417 °C measurement due to the lower losses of the formed nitrate. At both temperatures, there was only a slight desorption of weakly bound NO_x_ when the NO_2_ dosage was switched off, which also did not change the almost linear load-dependent behavior of the dielectric losses. Although losses increased significantly during NO_x_ loading, it was difficult to derive $x_{\mathrm{Ba},\mathrm{NOx}}$ due to the low signal-to-noise ratio, as the noise amounted to around 25% of the maximum loss change. Regarding permittivity, as already assumed from the individual measurement in Figure 8, at 417 °C, no clear conclusion can be drawn about the nitrogen oxide loading. When measuring at lower temperatures, the permittivity increased slightly over large areas of the loading phase. However, during this measurement, shortly after the start of NO_x_ loading, a strong change in the measured permittivity occurred, caused by a disturbance of the gas volume flow, resulting in an inaccuracy in the temperature measurement required for the MCP evaluation.

In general, due to the limited storage capacity of barium carbonate, it would be nearly impossible for a sensor system to detect changes in material properties caused by nitrogen oxide exposure. Therefore, in order to develop a sensitive NO_x_ dosimeter, a material must be found that exhibits a significantly larger change in complex permittivity due to an increased storage rate.

#### 4.2.2. Barium-Coated Alumina

We investigate whether the barium-coated material is suitable for such applications due to its significantly higher surface area. Since platinum particles were added to this material as oxidation catalysts, the storage material will not only be exposed to NO_2_ but also to NO. The purpose of these experiments is to determine the maximum amount that can be stored, the temperature-dependent behavior of the weakly bound NO_x_ storage, and whether the change in the dielectric properties is solely dependent on the amount of stored NO_x_. To analyze the latter point in more detail, the dosed nitrogen oxide concentration gradually increased from 100 over 200 to 300 ppm during the loading phases. The thermal desorption phase followed the same sequence as in the barium carbonate experiments.

**Figure 10:**
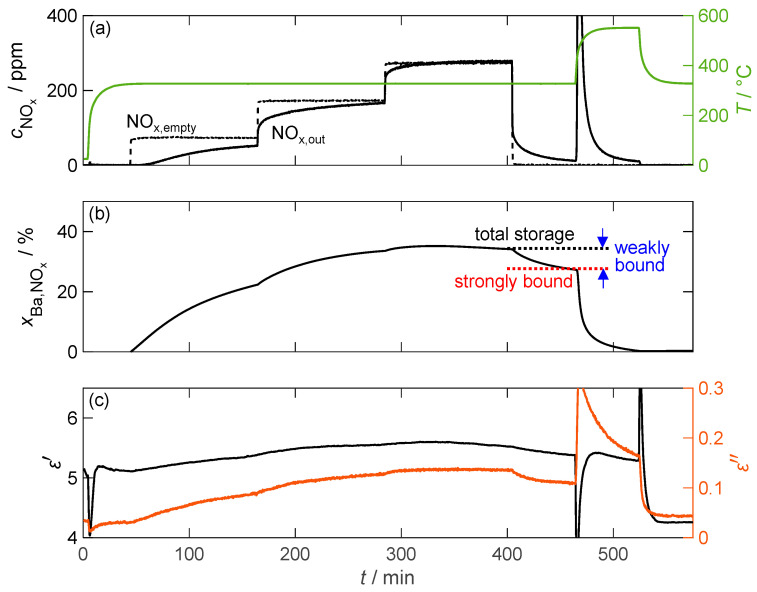
The storage behavior of the storage material with 16.9 wt.% barium carbonate coating over time during exposure to NO_2_; (**a**) the NO_x_ concentration $c_{\mathrm{NOx}}$ without (dashed line) and with (solid line) the storage material measured by FTIR downstream of the MCP setup as well as the temperature *T* of the storage material (green line); (**b**) the calculated storage utilization $x_{\mathrm{Ba},\mathrm{NOx}}$ based on an integration of the measured nitrogen oxide concentration; (**c**) the permittivity $\varepsilon^{\prime }$ and dielectric losses $\varepsilon^{\prime \prime }$ of the storage material.

Figure 10 exemplifies the storage behavior of the 16.9 wt.% barium carbonate-coated material when exposed to NO_2_ at a temperature of 330 °C. In contrast to the pure barium carbonate sample, significantly more nitrogen oxide is stored. As a result, the breakthrough of nitrogen oxide through the powder sample bed (downstream of the powder) can be detected only 15 min after the beginning of dosing. This is reflected in the storage capacity determined in an integrative manner, which increases by up to 35% of the available barium, which is more than 200 times that of pure barium carbonate. This increase in storage capacity should result in a similar increase in sensitivity of the NO_x_ dosimeter signal due to the linear relationship with the dielectric properties. However, the slightly increased amount of weakly bound NO_x_ compared to pure carbonate poses a problem for the dosimeter application because 20% of the stored NO_x_ is released after the NO_2_ dosing is terminated. Consequently, this effect implies that the dosimeter signal can only be interpreted if the gas concentration is known, which can be determined by the temporal differentiation of the measured material parameters [61]. The increase in the NO_2_ concentration also has a small effect on the storage capacity. At the end of the 200 ppm dosage, no additional NO_x_ can be adsorbed. However, increasing the concentration to 300 ppm results in a further 2% increase in the storage capacity. To show the exact influence on the operation of a dosimeter based on this material, further investigations would have to be carried out, focusing on the storage capacity at low NO_x_ concentrations down to a few ppm.

As expected from the storage behavior, the dielectric properties of the storage material exhibit significantly larger changes than those of pure barium carbonate. However, the permittivity cannot be accurately determined due to a temporal drift of the underlying resonant frequency that occurs before the start of the loading phase and continues until after thermal desorption. After this point, a constant permittivity could be measured. This behavior was observed in all measurements conducted. The dielectric losses, however, did not face this problem and increased by a factor of more than 4, from 0.03 to 0.14. Although the increase factor in $\varepsilon^{\prime \prime }$ is only twice that of pure barium carbonate due to the increased losses in the unloaded state, it is accompanied by a significantly improved signal-to-noise ratio. Both material parameters also reflect the gradual increase in NO_x_ storage due to the increased NO_2_ dosage.

**Figure 11:**
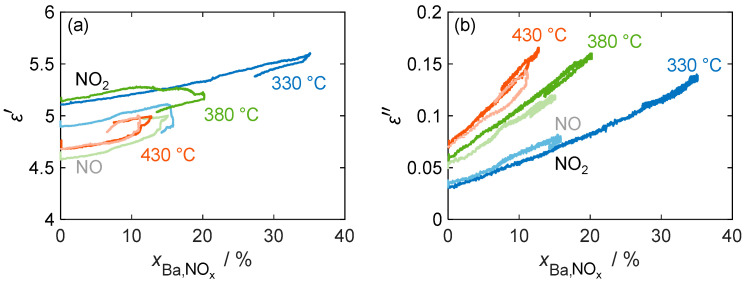
(**a**) The permittivity $\varepsilon^{\prime }$ and (**b**) dielectric losses $\varepsilon^{\prime \prime }$ of the storage material with 16.9 wt.% barium carbonate coating during NO (pale color tone) as well as NO_2_ (bold color) storage at different temperatures over the calculated storage utilization $x_{\mathrm{Ba},\mathrm{NOx}}$.

Figure 11 confirms that the material parameters depend only on the stored NO_x_ and not on the actual NO_x_ concentration by showing a linear relation of $\varepsilon^{\prime }$ and $\varepsilon^{\prime \prime }$ versus the storage utilization $x_{\mathrm{Ba},\mathrm{NOx}}$ across different temperatures (330 °C, 380 °C, and 430 °C). The diagram not only includes the material properties during NO_2_ dosing, but also shows a comparison with the material behavior during NO incorporation, where NO has to be oxidized with the aid of the platinum catalyst. In general, the linear relationship between the material properties and the amount of stored NO_x_ (expressed by $x_{\mathrm{Ba},\mathrm{NOx}}$) holds for all temperatures and stored NO_x_ species. Due to the permanent temporal drift of the resonant frequency and, therefore, of the resulting permittivity in all measurements, no clear conclusion can be drawn about the temperature and nitrogen oxide-dependent behavior of the permittivity.

These issues, however, did not affect the independent measurement of the quality factor and the resulting dielectric losses. In addition to the linear increase in $\varepsilon^{\prime \prime }$, none of the measurements showed hysteresis behavior during the desorption of the weakly bound NO_x_. The sensitivity of the dielectric losses increases with the temperature from 0.31%/%_Ba,NOx_ at 330 °C to 0.73%/%_Ba,NOx_ at 430 °C (calculated by linear regression based on NO_2_ storage). This is consistent with the characterization of the base materials in Figure 7, where a similar increase in the loss difference between nitrate and carbonate was measured.

The comparison of NO and NO_2_ storage shows no significant difference in loss sensitivity, as NO is oxidized by the oxygen present in the gas stream at the catalytically active platinum sites and stored as NO_2_. However, the maximum storage capacity for dosed NO is significantly reduced, especially at low temperatures. This reduction can be explained by the reduced catalytic activity of the platinum sites at temperatures below 350 °C [45]. Therefore, not all of the dosed NO can be oxidized to NO_2_, resulting in a reduced partial pressure of NO_2_. As previously determined by the variation in the dosed nitrogen oxide concentration, this affects the maximum storage quantity.

**Figure 12:**
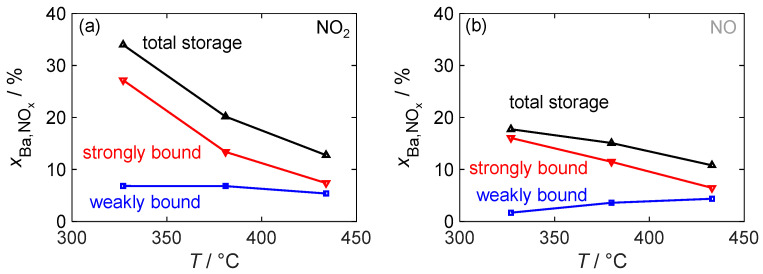
The temperature-dependent NO_x_ storage behavior of the material with 16.9 wt.% barium carbonate coating for (**a**) NO_2_ dosing and for (**b**) NO dosing; besides the total storage utilization $x_{\mathrm{Ba},\mathrm{NOx}}$, the proportions of strongly and weakly bonded NO_x_ are also shown.

Since the storage capacity and, in particular, the amount of strongly and weakly bound NO_x_ affect the functionality of the dosimeter, they were analyzed in more detail as a function of the material temperature. Figure 12 depicts the total storage capacity, defined as the value of $x_{\mathrm{Ba},\mathrm{NOx}}$ at the end of the NO_x_ storage phase, during NO_2_ and NO exposure as well as strongly and weakly bound NO_x_. The definitions of these terms are visually depicted in Figure 10.

With the NO_2_ dosage, the resulting total storage capacity decreases significantly with the temperature. However, compared to pure barium carbonate, a significantly higher mass storage capacity can be achieved even at the highest investigated temperature of 430 °C. This decrease in storage capacity is solely due to a reduced ability to form strongly bound nitrogen oxides. In contrast, the absolute amount of weakly bound NO_x_ remains constant. The storage behavior during NO dosage generally follows a similar pattern. However, at the lowest investigated temperature of 330 °C, the amounts of both weakly and strongly bound NO_x_ are reduced. The limitation caused by the oxidation of NO to NO_2_ leads to an almost halved total storage capacity. This also means that, in contrast to NO_2_ dosing, the amount that can be stored as weakly bound NO_x_ decreases at low temperatures, so that at 330 °C, over 90% of the stored NO_x_ is strongly bound. At higher temperatures, the storage capacities for NO and NO_2_ become similar. At 430 °C, their behavior is almost identical.

**Figure 13:**
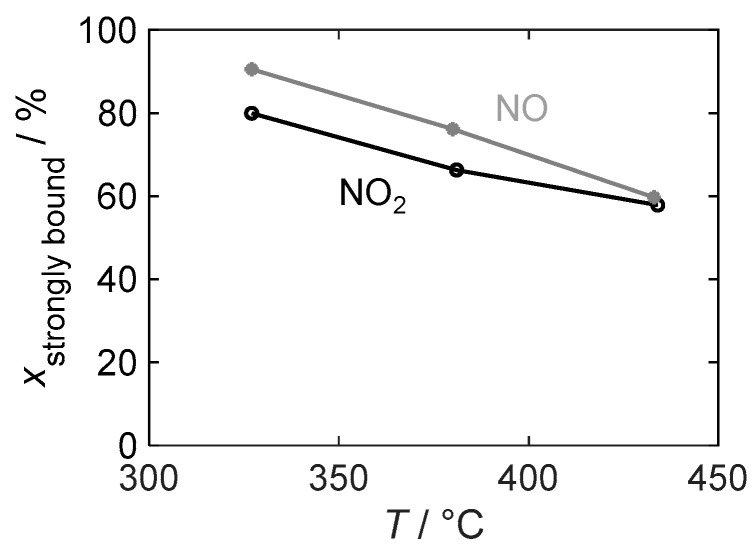
The temperature-dependent proportion of strongly bound nitrogen oxide $x_{\mathrm{strongly}\;\mathrm{bound}}$ relative to the total stored NO_x_ in the material with 16.9 wt.% barium carbonate coating during NO_2_ and NO exposure.

Figure 13 illustrates the temperature dependence of the binding stability of the stored gas by showing the strongly bound NO_x_ as a percentage of the total stored amount, which is relevant for the dosimeter behavior of a sensor. At all investigated temperatures, the strongly bound fraction is higher for the detection of NO than for NO_2_ and is always above 55%. However, the difference decreases with an increasing temperature and is almost negligible at 430 °C.

To operate a dosimeter using this material, a trade-off must therefore be made between the signal strength associated with the change in dielectric material properties due to stored NO_x_, the absolute storage capacity, and the nitrogen oxide-dependent sensor response. Although a significant change in dielectric properties occurs at temperatures above 400 °C, and NO and NO_2_ exhibit similar storage behavior, operation at 350 °C offers the advantage of an increased maximum storage capacity and only a small amount of weakly bound NO_x_, which would interfere with the evaluation of the dosimeter signal. Sensor operation at either temperature may be suitable depending on the intended application. If the sensor is to be operated in an environment with NO as well as NO_2_ concentrations, a higher temperature is required. On the other hand, if only the presence of NO or NO_2_ is expected, a higher storage capacity at lower temperatures may allow the sensor to operate longer without the need for regeneration.

## 5. Conclusions

In response to the growing demand for accurate and efficient NO_x_ monitoring, this study started to develop an RF-based NO_x_ gas dosimeter by focusing on the LTCC-based sensor substrates as well as the barium-based materials used for NO_x_ storage. By applying the Microwave Cavity Perturbation method, the dielectric properties of these materials were analyzed. Knowledge about these properties is necessary to support the design process of the dosimeter’s development regarding an improved sensor signal.

In general, the investigations showed that a NO_x_ dosimeter must be operated at high temperatures to allow for a kinetically unrestricted NO_x_ uptake. Therefore, a potential sensor substrate for such an application would be LTCC, which also offers several manufacturing advantages, such as the possibility of a multilayer sensor design that includes a heating structure. DuPont GreenTape 9K7, specifically designed for RF applications with reduced dielectric losses at room temperature, was compared to DuPont GreenTape 951 in a temperature range of up to 600 °C. Both exhibited a non-linear increase in dielectric loss with the temperature, which is the primary contributor to transmission line attenuation. At temperatures above 500 °C, the dielectric losses of DuPont 9K7 were found to exceed those of DuPont 951. However, within the main operating temperature range for NO_x_ incorporation between 300 and 450 °C, the 9K7 material is still the preferred option.

In addition, this study focused on barium-based NO_x_ storage materials and their gas dosimeter signal, which is derived from the change in dielectric properties as the barium carbonate is converted to nitrate during NO_x_ storage. The difference in dielectric properties between pure barium carbonate and nitrate increases with temperature, resulting in a stronger dosimeter signal at higher temperatures. However, due to its low surface area and, therefore, limited storage capability, pure barium carbonate cannot be used as a gas dosimeter material. This issue could be addressed by coating high surface alumina with barium carbonate. The addition of a catalytic platinum coating not only facilitated the storage of both NO_2_ and NO gases, but also revealed that dielectric properties are solely influenced by the amount of stored NO_x_ (and not by the applied NO_x_ concentration). While the storage characteristics were similar for NO_2_ and NO with a constant amount of weak bonds and a decrease in strong bonds with temperature, the maximum storage capacity differed at low temperatures due to the limited catalytic effect of platinum, resulting in a lower uptake of NO. However, at the highest temperature studied, 430 °C, there was no difference in the storage behavior between the two nitrogen oxides.

Our research successfully identified the critical parameters that influence the design and operation strategy of RF-based NO_x_ dosimeters by elucidating the dielectric behavior of LTCC substrates and barium-based NO_x_ storage materials. Based on these results, a functioning NO_x_ dosimeter can now be developed and analyzed in future studies.

## Figures and Tables

**Table 1:** The shrinkage of the LTCC green tapes DuPont 951 and 9K7 in the layer plane (*x*,*y*-direction) as well as along the layer thickness (*z*-direction); besides the measured values, the data sheet values are also given.

LTCC	*x*,*y*-Shrinkage		*z*-Shrinkage	
	Measured	In the Literature	Measured	In the Literature
9K7	7.8% ± 0.3%	9.1% ± 0.3%	12.2% ± 1.2%	11.8% ± 0.5%
951	12.4% ± 0.2%	12.7% ± 0.3%	16.9% ± 1.6%	15.0% ± 0.5%

**Table 2:** BET analysis of pure BaCO_3_ and highly porous alumina coated with different amounts of barium carbonate.

Material	BET Surface/m^2^/g
BaCO_3_	1.7
5.6 wt.% BaCO_3_ @ Al_2_O_3_	63
11.2 wt.% BaCO_3_ @ Al_2_O_3_	57
16.9 wt.% BaCO_3_ @ Al_2_O_3_	46
γ-Al_2_O_3_	72

## Data Availability

All relevant data presented in the article are stored according to institutional requirements and, as such, are not available online. However, all data used in this paper can be made available upon request to the authors.
